# Moderate alcohol consumption is associated with significant fibrosis progression in NAFLD

**DOI:** 10.1097/HC9.0000000000000003

**Published:** 2023-01-10

**Authors:** Julia Blomdahl, Patrik Nasr, Mattias Ekstedt, Stergios Kechagias

**Affiliations:** Department of Gastroenterology and Hepatology and Department of Health, Medicine, and Caring Sciences, Linköping University, Linköping, Sweden

## Abstract

The effect of moderate alcohol consumption on NAFLD histology is disputed. Assessment of alcohol consumption is commonly performed with interview or questionnaires. Phosphatidylethanol (PEth) in blood is a highly sensitive and specific alcohol biomarker, which only forms in the presence of ethanol. PEth has hitherto not been evaluated in longitudinal NAFLD studies. This study aimed to examine the impact of moderate alcohol consumption on histologic progression and evaluate the utility of PEth in NAFLD. NAFLD patients with serial biopsies were reviewed for inclusion in the study. At baseline, all patients reported alcohol consumption <140 g/week. Anthropometric and biochemical measurements were performed at baseline and follow-up. Alcohol consumption was assessed thoroughly at follow-up with clinical interview, the Alcohol Use Disorder Identification Test-Consumption (AUDIT-C) questionnaire, and analysis of PEth in whole blood. Eighty-two patients were included. Mean follow-up time was 17.2 years (SD±6.0). Patients with significant fibrosis progression (defined as progression of ≥2 stages or development of cirrhosis-related complications) reported higher alcohol consumption and had significantly higher PEth. Consumption >66–96 g/week (but <140 g) (i.e. moderate alcohol consumption) was associated with increased risk of significant fibrosis progression compared with no or low consumption. PEth ≥48 ng/mL and binge drinking showed the highest risk for significant fibrosis progression (aOR: 5.9; 95% CI: 1.6–21.4) and aOR: 5.1; 95% CI: 1.4–18.1, respectively). NAFLD patients consuming moderate amounts of alcohol are at increased risk for significant fibrosis progression and development of cirrhosis-related complications. PEth is a potential biomarker to assess harmful alcohol consumption in NAFLD. Patients reporting moderate consumption or exhibiting PEth ≥48 ng/mL should be advised to reduce alcohol consumption.

## INTRODUCTION

NAFLD is the most common chronic liver disease, affecting ~25% of the adult population worldwide.^1^ Even though a majority of NAFLD patients never experience liver-related symptoms, it is estimated that ~5%–10% will develop cirrhosis-related complications with risk of death or need for liver transplantation.^2^,^3^ Although inflammation and hepatocellular injury (i.e. NASH) is considered a more progressive form of NAFLD, follow-up studies have shown that hepatic fibrosis remains the strongest independent predictor of clinical outcome.^4^,^5^

In the Western world, alcohol overconsumption is the leading cause of decompensated cirrhosis.^6^ NAFLD and alcohol-related liver disease share histopathological characteristics, and the two conditions are separated by the amount of alcohol consumed. The maximum weekly consumption of alcohol in NAFLD is set at 210 g for males and 140 g for females.^7^ There are no guidelines on how to best measure alcohol consumption in NAFLD patients. Usually, clinical interview and/or self-reporting is used. However, there are also various questionnaires available, such as the validated and commonly used Alcohol Use Disorder Identification Test (AUDIT).^8^ A shorter version of AUDIT [i.e. AUDIT-Consumption (AUDIT-C)] is also available, which focuses on consumption and frequency rather than on symptoms of dependence.^9^ Currently, few direct biomarkers of alcohol consumption are available.^10^ The most easily accessible is phosphatidylethanol (PEth) in blood, which only forms in the presence of ethanol. It has high sensitivity (94% to 100%) and specificity (100%), is analyzed from whole blood, and can detect alcohol use the previous 2–4 weeks.^10^ A cut-off value between moderate consumption and overconsumption of alcohol has been suggested at 210 ng/mL (0.3 µmol/L). A level >500 ng/mL (0.7 µmol/L) is considered as a previous prolonged period of alcohol overconsumption.^11^

Alcohol consumption of 1–2 drinks per day has previously been shown to be associated with reduced risk of cardiovascular disease^12^ and improved insulin sensitivity 13—traits that are frequently seen in NAFLD patients. Some studies have investigated cardiovascular outcomes in NAFLD patients with moderate alcohol consumption.^14^–^16^ They indicate that light alcohol consumption (i.e. 0–9 g/d) decreases the risk of cardiovascular disease compared to lifetime abstainers. Moreover, Janjua et al^16^ recently reported reduced risk of hospitalization from cardiovascular disease in NAFLD patients with moderate alcohol consumption (8–21 drinks per week) compared to light consumption (1–7 drinks per week). Hajifathalian et al^17^ showed reduced all-cause mortality in subjects consuming 0.5–1.5 drinks per day but significantly increased overall mortality in those consuming ≥1.5 drinks per day. However, it is disputed if alcohol has a favorable or detrimental effect on the liver in NAFLD. To date, there are fifteen studies of biopsy-proven NAFLD that have investigated the impact of moderate alcohol consumption on the liver.^18^–^32^ In general, the combined results of these studies are conflicting, and hence there are no specific recommendations concerning light to moderate alcohol consumption in NAFLD patients. This study aimed to examine the impact of moderate alcohol consumption on histologic progression in patients with NAFLD. Moreover, we evaluated the utility of PEth to assess alcohol consumption in NAFLD.

## PATIENTS AND METHODS

### Study design and participants

In this cohort study, included subjects were initially referred to the Department of Gastroenterology and Hepatology, University Hospital, Linköping, between 1988 and 2017, because of prolonged (>6 mo) elevation of serum aminotransferases or a finding of steatosis on imaging. Upon inclusion, patients underwent clinical interview and examination, including medical and pharmaceutical history, blood tests, and liver biopsy. All included patients reported alcohol consumption <140 g/week and were diagnosed with NAFLD after exclusion of secondary causes of steatosis.

The total cohort consists of ~300 patients and the majority have been re-evaluated at least once after inclusion. At follow-up, participants were offered clinical and biochemical re-evaluation and a repeat liver biopsy. The participants also completed a self-report questionnaire (AUDIT-C). Liver biopsy was not offered at follow-up if the patient had previously been diagnosed with cirrhosis.

Inclusion criteria for this study were:

- Liver biopsy performed at follow-up or a clinical diagnosis of cirrhosis.
- Available whole blood from follow-up stored at −80°C (for the analysis of PEth).
- Available self-report questionnaire (AUDIT-C) from the follow-up visit.

### Data collection at follow-up

For each patient, a standardized case report form was completed covering previous and current medical history, including current and previous medication. The patient underwent measurement of blood pressure and general physical examination, as well as anthropometric measurements of weight, height, and waist circumference. Blood tests were drawn after an overnight fast. Blood samples (whole blood, serum, and plasma) were also stored in −80°C freezers for later use.

### Questionnaires and assessment of alcohol consumption at follow-up

All patients filled out the AUDIT-C questionnaire independently and without interference from the researchers. The AUDIT-C consists of 3 questions: (1) How often do you have a drink containing alcohol? (2) How many drinks containing alcohol do you have on a typical day when you are drinking, and (3) How often do you have 6 or more drinks on 1 occasion?.^9^ Each patient was carefully interviewed about current and previous alcohol consumption and the reported consumption was transformed into standard drinks. Calculations of average weekly ethanol consumption were made from data obtained from the AUDIT-C questionnaire and the clinical interview. One standard unit of alcohol was equal to 12 g of ethanol.^33^

### Analysis of PEth

PEth was analyzed from previously stored whole blood at follow-up. The samples were thawed and then analyzed by the Clinical Pharmacology Laboratory, Region Östergötland. PEth was measured using the LC-MS/MS (liquid chromatography with tandem mass spectrometry) method.^34^ The limit of quantification was at 18 ng/mL (0.025 µmol/L). Analysis of PEth was made blinded to patient data.

### Liver biopsies

All liver biopsies were performed percutaneously with ultrasound guidance using a 1.6 mm Biopince needle (Argon Medical Devices, Dallas, TX). Biopsies were performed within 3 months of clinical evaluation, both at baseline and follow-up. Histological parameters were reviewed by an experienced liver pathologist and graded according to Kleiner et al.^35^ The diagnosis of NASH was defined using the fatty liver inhibition progression algorithm.^36^

### Fibrosis progression

Study participants were divided into 2 groups depending on significant fibrosis progression during follow-up. Significant fibrosis progression was defined as change in fibrosis stage by 2 or more fibrosis stages. Unchanged, regression or progression by 1 stage was considered as absence of significant fibrosis progression. Patients who developed cirrhosis-related complications during follow-up were considered to have significant fibrosis progression, irrespective of fibrosis stage at baseline. Cirrhosis-related complications were defined as development of ascites, moderate or large esophageal varices or variceal hemorrhage, hepatic encephalopathy, or hepatocellular carcinoma.

### Statistical analysis

Continuous variables are presented as mean±SD and binominal variables as percentages. Categorical variables are presented as median with interquartile range. Baseline and follow-up characteristics were compared using paired *t* test for continuous variables and the Fisher exact test for categorical variables. Correlations were calculated using Pearson *r* or Spearman rho (ρ) when applicable. Differences between 2 groups were evaluated using the Student *t* test or Mann-Whitney *U* test for variables not normally distributed. For categorical variables, Fisher exact test was used. Binary logistic regression models were performed. Age, sex, body mass index, weight change (i.e. weight difference between baseline and follow-up), diagnosis of type 2 diabetes mellitus (T2DM), NASH at baseline biopsy, and alcohol consumption (assessed by AUDIT-C, interview, and PEth) were all tested separately in univariable analyses for development of fibrosis progression ≥2 stages or cirrhosis-related complications (Model 1). We attained different intervals of alcohol consumption from the logistic regression model. Consumption assessed by AUDIT-C was divided into 3 groups: (1) 0–2.99 g/week; (2) 3–65.99 g/week; (3) ≥66 g/week. Consumption assessed by interview was divided into 3 groups: (1) 0–2.99 g/week; (2) 3–96 g/week; (3) ≥96.1 g/week. Average PEth-value was divided into 2 groups based on optimal cut-off derived from receiver operating characteristic analysis; (1) <48 ng/mL; (2) ≥48 ng/mL. A second model (Model 2) was performed with a stepwise-forward approach to adjust for any significant covariables by using *p*<0.10 as a threshold of significance regarding age, sex, weight change, body mass index, T2DM, and NASH. Lastly, a third model (Model 3) was performed selecting important clinical parameters (i.e. age, sex, weight change, T2DM, and NASH), irrespective of their significance levels. A *p* value of <0.05 was considered statistically significant. All statistical analyses were performed using IBM SPSS Statistics for Windows, version 26.0 (IBM Corp., Armonk, NY).

### Ethical considerations

All participants gave written informed consent. The study was conducted in full accordance with the ethical guidelines of the 1975 Declaration of Helsinki. The regional ethics committee at the University Hospital in Linköping approved the study design (02-454, amendments: 2011/468-32, 2012-229-32 and 2013/72-32).

## RESULTS

### Patient characteristics

Eighty-two patients were included. Mean follow-up time was 17.2 years (SD±6.0, range: 3.3–32.8 y) with 1409 person-years of follow-up. Patient characteristics from baseline and follow-up are presented in Table 1. Participants were predominantly overweight males with hypertension. Only a few (12.2%) were diagnosed with T2DM at baseline, but a majority had been diagnosed at follow-up (59.8%). Alanine aminotransferase levels were significantly lower at follow-up, and thus aspartate aminotransferase/alanine aminotransferase ratio was significantly higher (Table 1).

**TABLE 1:** Patient characteristics

	Baseline (n=82)	Follow-up (n=82)	*p*
Age (years)	47.3 (±11.2)	64.5 (±10.7)	**<0.01**
Sex (% male)	72 (n=59)	72 (n=59)	NA
BMI (kg/m^2^)	28.1 (±3.9)	29.0 (±4.4)	**0.02**
Weight change (kg)^a^	NA	2.0 (±9.3)	NA
Diagnosis of T2DM (%)	12.2 (n=10)	59.8 (n=49)	**<0.05**
Hypertension (%)	63.4 (n=52)	87.8 (n=72)	**<0.05**
ALT (U/L)	83.4 (±48.0)	54.6 (±36.0)	**<0.01**
AST (U/L)	45.6 (±21.6)	40.2 (±19.8)	0.07
AST/ALT ratio	0.6 (±0.2)	0.9 (±0.4)	**<0.01**
ALP (U/L)	66.0 (±36.0)	72.0 (±48.0)	0.05
Bilirubin (mg/dL)	0.64 (±0.33)	0.74 (±0.30)	**0.01**
Albumin (g/L)	41.6 (±3.4)	40.4 (±4.1)	**0.03**
Hemoglobin (g/L)	146.9 (±9.9)	144.7 (±13.7)	0.96
Platelet count (10^9^/L)	223.6 (±53.1)	227.6 (±63.9)	0.96
Prothrombin (INR)	1.0 (±0.1)	1.0 (±0.1)	**<0.01**
Creatinine (mg/dL)	1.01 (±0.22)	1.01 (±0.25)	0.97
Ferritin (µg/L)	210.9 (±171.0)	196.7 (±170.9)	0.47
Fasting glucose (mg/dL)	108.0 (±37.8)	135.0 (±43.2)	**<0.01**
HOMA-IR	NA	8.56 (±13.04)	NA
Fibrosis stage (IQR)	1 (0–2)	1.5 (0–3)	**<0.001**
Steatosis grade (IQR)	2 (1–3)	2 (1–2)	0.15
Lobular inflammation (IQR)	0	0	**0.03**
Ballooning (IQR)	0	0 (0–0.75)	0.26
NASH (%)	9.0 (n=7)	8.3 (n=6)	0.31

^a^Weight change between baseline and follow-up.

Abbreviations: NA indicates not applicable; BMI, body mass index; T2DM, type 2 diabetes mellitus; ALT, alanine aminotransferase; AST, aspartate aminotransferase; AST/ALT ratio; aspartate aminotransferase/alanine aminotransferase ratio; ALP, alkaline phosphatase; HOMA-IR, Homeostatic Model Assessment for Insulin Resistance; INR, international normalized ratio.

Bold values indicate statistical significance at the *p* < 0.05 level.

### Assessment of alcohol consumption

All participants were carefully interviewed about their alcohol consumption at baseline and all subjects reported alcohol consumption <140 g/week. No questionnaires, whole blood, or exact quantification of weekly consumption were available from baseline. At follow-up, all patients (n=82) were interviewed about their average weekly alcohol consumption, filled out the AUDIT-C questionnaire, and had blood drawn for analysis of PEth. Mean weekly alcohol consumption at follow-up was 28.6 (±32.2, range: 0–108) and 32.1 (±38.8, range: 0–132) grams/week assessed by AUDIT-C and clinical interview, respectively. Average PEth-value was 55.0 (±86.9) ng/mL (Table 2). Twenty-one participants (25.6%) reported binge drinking (defined as ≥5 standard units for males or ≥4 standard units for females of alcohol per occasion, at least once per month). A subset of patients (n=53) had an additional question attached to their AUDIT-C questionnaire. They were asked to account for if their consumption pattern had changed since baseline. The majority (57%) stated no change in consumption pattern over time, while 32% stated only minor changes (minor increase or decrease). Only 6 participants reported a significant reduction of consumption [mean weekly alcohol consumption at follow-up of 14.6 g/wk (assessed by AUDIT-C)]. Baseline characteristics of participants with no or minor changes in consumption showed a higher rate of hypertension and lower ALP levels compared with the remaining cohort (Supplementary Table 1, http://links.lww.com/HC9/A3). Although baseline fibrosis stage was lower, and baseline NASH less frequent in participants with no or minor changes in consumption over time compared to the remaining cohort, there was no significant difference in the rate of fibrosis progression at follow-up between the 2 groups (*p*=0.13) (Supplementary Table 1, http://links.lww.com/HC9/A3). Furthermore, no participant reported current or previous consumption of >140 g/week, nor that their consumption pattern had significantly increased during follow-up. All 3 methods of assessing alcohol consumption (AUDIT-C, interview, and analysis of PEth) correlated significantly (*r=*0.897 between AUDIT and interview; ρ=0.738 between AUDIT and PEth; ρ=0.716 between interview and PEth, all *p*<0.05).

**TABLE 2:** Alcohol consumption and average PEth-value

	No progression (n=60)	Significant fibrosis progression (n=22)	*p*	Whole cohort (n=82)
Alcohol consumption (AUDIT-C) (g/wk)	25.8 (±31.9)	36.2 (±34.9)	0.15	28.6 (±32.2)
Alcohol consumption (interview) (g/wk)	27.5 (±34.5)	44.5 (±47.3)	0.14	32.1 (±38.8)
PEth-value (ng/mL)	43.6 (±74.1)	86.3 (±111.1)	**0.02**	55.0 (±86.9)
Binge drinking (%)	18.3 (n=11)	45.5 (n=10)	**0.02**	25.6 (n=21)

Abbreviations: AUDIT-C indicates Alcohol Use Disorder Identification Test-Consumption; PEth, phosphatidylethanol.

Bold values indicate statistical significance at the *p* < 0.05 level.

### Histology and fibrosis progression

All patients (n=82) underwent liver biopsy at baseline and 89% (n=73) at follow-up. Nine patients did not undergo a second biopsy due to development of cirrhosis (n=6) or cirrhosis-related complications during follow-up [development of ascites (n=1), moderate or large esophageal varices or variceal hemorrhage (n=1), or hepatocellular carcinoma (n=1)]. They were considered to have fibrosis stage 4 in the analyses. Four participants had missing data on grade of ballooning and lobular inflammation in the baseline biopsies. Three of those also had missing data on steatosis grade and fibrosis stage. However, all 3 had fibrosis stage 0 or 1 at follow-up and were considered as having no fibrosis progression in the analyses. Median grade of steatosis and ballooning was similar at baseline and follow-up. Median grade of lobular inflammation progressed significantly at follow-up due to 11 patients (14.1%) progressing from grade 0 to grade 2 (however, median grade remained 0 at both baseline and follow-up, see Table 1). Median fibrosis stage at baseline was 1 (0–2) and progressed significantly at follow-up to 1.5 (0–3), *p*<0.001. Furthermore, 12 of the participants (14.6%) had cirrhosis at follow-up [biopsy-proven (n=9), diagnosed due to development of cirrhosis-related complications (n=3)]. Twenty-two participants (26.8%) progressed 2 or more fibrosis stages or had developed cirrhosis-related complications at follow-up (Table 3). Of those, 19 participants progressed in fibrosis stage, and 3 participants developed cirrhosis-related complications. Baseline characteristics of the participants with significant fibrosis progression were similar to those without progression (Table 3).

**TABLE 3:** Baseline characteristics according to fibrosis progression at follow-up

	No progression (n=60)	Significant fibrosis progression (n=22)	*p*
Age (y)	47.6 (±11.0)	46.5 (±11.9)	0.70
Sex (% male)	70.7 (n=42)	77.3 (n=17)	0.59
BMI (kg/m^2^)	28.0 (±4.1)	28.4 (±3.2)	0.66
Diagnosis of T2DM (%)	10.0 (n=6)	18.2 (n=4)	0.45
Hypertension (%)	70.6 (n=36)	88.9 (n=16)	0.20
ALT (U/L)	80.4 (±48.0)	92.4 (±54.0)	0.31
AST (U/L)	44.4 (±24.0)	49.2 (±24.0)	0.40
AST/ALT ratio	0.6 (±0.2)	0.6 (±0.1)	0.43
ALP (U/L)	66.0 (±42.0)	60.0 (±30.0)	0.46
Bilirubin (mg/dL)	0.62 (±0.23)	0.71 (±0.51)	0.29
Albumin (g/L)	41.6 (±3.5)	41.5 (±2.9)	0.85
Hemoglobin (g/L)	146.8 (±10.1)	147.3 (±9.5)	0.87
Platelet count (10^9^/L)	226.1 (±57.0)	216.5 (±40.7)	0.49
Prothrombin (INR)	1.0 (±0.1)	1.0 (±0.1)	0.29
Creatinine (mg/dL)	1.00 (±0.23)	1.04 (±0.18)	0.41
Ferritin (µg/L)	213.6 (±188.7)	204.1 (±119.4)	0.83
Fasting glucose (mg/dL)	108.0 (±36.0)	109.8 (±43.2)	0.88
Fibrosis stage (IQR)	1 (0–2)	0 (0–1)	0.17
Steatosis grade (IQR)	2 (1–3)	3 (2–3)	0.29
Lobular inflammation (IQR)	0	0 (0–0.25)	0.46
Ballooning (IQR)	0	0	0.64
NASH (%)	10.7 (n=6)	4.5 (n=1)	0.67

Abbreviations: BMI, body mass index; T2DM, type 2 diabetes mellitus; ALT, alanine aminotransferase; AST, aspartate aminotransferase; AST/ALT ratio; aspartate aminotransferase/alanine aminotransferase ratio; ALP, alkaline phosphatase; INR, international normalized ratio.

### Alcohol consumption and histological progression

Average PEth-value was significantly higher in patients with significant fibrosis progression (86.3±111.1 vs. 43.6±74.1 ng/mL, *p*=0.02) (Table 2). Average weekly consumption was higher, but not significantly different, in those with significant fibrosis progression compared to those with no progression as assessed by AUDIT-C (36.2±34.9 vs. 25.8±31.9 g/wk, *p*=0.15), as well in assessment with interview (44.5±47.3 vs. 27.5±34.5 g/wk, *p*=0.14) (Table 2). Ten participants (45.5%) reported binge drinking ≥1 per month in the significant fibrosis progression group compared with 11 (18.3%) in those without significant fibrosis progression (*p*=0.02). Only a small number of patients had progressed ≥1 grade of steatosis, lobular inflammation, and ballooning (n=12, 11, and 18, respectively) at follow-up. None of these variables showed significant associations with alcohol consumption or PEth (data not shown).

### Predictors of fibrosis progression

Logistic regression analyses were performed to find predictors associated with fibrosis progression. In the univariable analysis (i.e. Model 1, Table 4), binge drinking was significantly associated with significant fibrosis progression [crude odds ratio (cOR): 3.71, 95% CI: 1.28–10.76, *p*=0.02]. Furthermore, continuous values of weight change, PEth, and alcohol consumption (assessed by interview or AUDIT-C) showed trends toward higher OR for significant fibrosis progression, but none reached level of significance. Different intervals of consumption and PEth-value were also put in the regression model. Alcohol consumption ≥66 g/week (assessed by AUDIT-C) showed a cOR of 5.43 (95% CI: 1.06–27.83) for significant fibrosis progression compared to consumption of 0–2.99 g/week (*p*=0.04). Alcohol consumption >96 g/week (assessed by clinical interview) also showed significantly higher cOR for significant fibrosis progression (cOR: 8.00, 95% CI: 1.16–55.26, *p*=0.04). Furthermore, a PEth-value ≥48 ng/mL showed a cOR of 4.18 (95% CI: 1.42–12.26, *p*=0.01). In the second model (i.e. Model 2, Table 4), different alcohol consumption parameters were adjusted for clinical and anthropometric variables that achieved a *p* value <0.10 (which only applied for weight change). After adjustment, PEth-value ≥48 ng/mL and binge drinking remained significantly associated with significant fibrosis progression (aOR: 4.05, 95% CI: 1.32–12.44 and 3.34, 95% CI: 1.09–10.29, respectively). In the third model (i.e. Model 3, Table 4), alcohol consumption (assessed by AUDIT-C, interview, and PEth) and binge drinking were adjusted for clinically relevant parameters (i.e. sex, age, weight change, T2DM, and NASH) irrespective of their status of univariable significance. Still, only PEth ≥48 ng/mL and binge drinking were statistically associated with increased odds of significant fibrosis progression (aOR: 5.90, 95% CI: 1.62–21.44 and 5.09, 95% CI: 1.43–18.14, respectively).

**TABLE 4:** Predictors of significant fibrosis progression

	Model 1^a^			Model 2^b^			Model 3^c^		
Variable	cOR	95% CI	*p*	aOR	95% CI	*p*	aOR	95% CI	*p*
Clinical parameters									
Sex (male)	1.46	0.47–4.56	0.52						
Age (y)	0.99	0.95–1.04	0.70						
BMI (kg/m^2^)	1.03	0.91–1.17	0.65						
Weight change (kg)	1.05	0.96–1.12	0.07						
T2DM (yes)	2.00	0.51–7.90	0.32						
NASH (yes)	0.40	0.05–3.50	0.41						
Weekly alcohol consumption:									
AUDIT-C (continuous)	1.01	1.00–1.03	0.20	1.01	0.99–1.03	0.28	1.01	0.96–1.03	0.17
AUDIT-C (0–2.99 g/wk)	(ref)			(ref)			(ref)		
AUDIT-C (3–65.9 g/wk)	2.42	0.61–9.58	0.21	2.07	0.51–8.42	0.31	2.17	0.42–11.24	0.36
AUDIT-C (≥66 g/wk)	5.43	1.06–27.83	**0.04**	4.78	0.86–26.55	0.07	7.01	0.93–52.95	0.06
Interview (continuous)	1.01	1.00–1.02	0.08	1.01	1.00–1.02	0.14	1.01	1.00–1.03	0.13
Interview (0–2.99 g/wk)	(ref)			(ref)			(ref)		
Interview 3–96 g/wk	2.31	0.59–8.99	0.23	2.08	0.52–8.24	0.30	2.10	0.40–10.88	0.38
Interview >96 g/wk	8.00	1.16–55.26	**0.04**	8.55	0.99–73.97	0.05	10.02	0.82–122.65	0.07
Direct alcohol marker									
PEth (continuous)	1.01	1.00–1.01	0.07	1.00	1.00–1.01	0.16	1.00	1.00–1.01	0.17
PEth (≥48 ng/mL)	4.18	1.42–12.26	**0.01**	4.05	1.32–12.44	**0.01**	5.90	1.62–21.44	**0.01**
Drinking pattern									
Binge drinking	3.71	1.28–10.76	**0.02**	3.34	1.09–10.29	**0.04**	5.09	1.43–18.14	**0.01**

^a^ Unadjusted estimates.

^b^ Adjusted for weight change.

^c^ Adjusted for weight change, sex, T2DM, age, and NASH.

Univariate and multivariate odds ratio for fibrosis progression ≥2 stages or development of cirrhosis-related complications.

Abbreviations: AUDIT-C indicates Alcohol Use Disorder Identification Test-Consumption; BMI, body mass index; PEth, phosphatidylethanol; T2DM, type 2 diabetes mellitus.

Bold values indicate statistical significance at the *p* < 0.05 level.

## DISCUSSION

The main finding in this study of NAFLD patients with serial biopsies, is that moderate alcohol consumption is associated with significant fibrosis progression. This is the first study evaluating PEth as a marker for alcohol consumption in long-term follow-up of NAFLD patients. PEth correlated well with alcohol consumption assessed with AUDIT-C and clinical interview. Values exceeding 48 ng/mL were strongly associated with fibrosis progression. In a previous cross-sectional study by our group,^32^ we evaluated the association between moderate alcohol consumption and the prevalence of advanced fibrosis in NAFLD. We reported that moderate alcohol consumption was associated with advanced liver fibrosis. Furthermore, we found that the prevalence of advanced fibrosis was more pronounced in patients with moderate alcohol consumption and concurrent T2DM. In the present longitudinal study, we extend our previous findings and show that moderate alcohol consumption not exceeding 140 g/week appears to be an important factor of fibrosis progression in NAFLD. Binge drinking or consumption exceeding 66 g/week assessed with AUDIT-C or 96 g/week assessed with clinical interview were the strongest factors for fibrosis progression.

Assessment of alcohol consumption with questionnaires or interview can be time-consuming as well as inaccurate, particularly in patients with liver disease who may be prone to neglect consumption of low amounts of alcohol. It has previously been reported that PEth is the biochemical marker that best reflects alcohol consumption in healthy volunteers.^37^ In the present study we show that PEth strongly correlates with low to moderate alcohol consumption in NAFLD patients and thus can be used to monitor alcohol consumption in these patients. Moreover, patients with values ≥48 ng/mL should be advised to abstain from alcohol, irrespective of reported consumption, since the likelihood of fibrosis progression is increased.

To our knowledge, 4 previous studies have evaluated the impact of moderate alcohol consumption on progression of liver disease in NAFLD patients.^20^,^21^,^26^,^29^ Two of them focused on hepatocellular carcinoma development and concluded that moderate alcohol consumption was a risk factor for hepatocellular carcinoma in NAFLD patients with advanced fibrosis.^21^,^29^ The other 2 evaluated histological progression and showed that binge drinking was a risk factor for fibrosis progression and that moderate alcohol consumption was associated with less reduction in steatosis and NASH.^20^,^26^ The results of our study are in line with these studies. On the other hand, the results from cross-sectional studies are contradictory. A meta-analysis by Wijarnpreecha et al^38^ consisting of 6 cross-sectional studies, reported a pooled OR of 0.51 for advanced fibrosis in moderate drinkers. Another recent meta-analysis by Wongtraktul et al^39^ included a total of 14 studies. Eight studies compared the risk for advanced fibrosis and modest alcohol consumption. In accordance with the findings of Wijarnpreecha and colleagues, they found a pooled OR of 0.59 for advanced fibrosis. However, they also reported an increased risk for hepatocellular carcinoma in NAFLD subjects consuming moderate amounts of alcohol. A major weakness of cross-sectional studies, including meta-analyses, is that conclusions on causality cannot be drawn. In the absence of the ability to perform randomized controlled studies on the impact of moderate alcohol consumption on NAFLD we must primarily rely on longitudinal retrospective studies. Clearly, in fatty liver disease alcohol consumption as well as metabolic syndrome are both negative prognostic factors affecting survival and morbidity.^40^

An interesting finding in our study is that significant fibrosis progression occurred in those consuming moderate amounts of alcohol despite that the other histological parameters (i.e. steatosis, lobular inflammation, and ballooning) were unaffected during follow-up. In fatty liver disease associated with more pronounced alcohol consumption, that is, alcohol-related liver disease, steatohepatitis characterized by a combination of each, steatosis, lobular inflammation, and hepatocellular ballooning is considered the most potent driver of fibrosis.^41^ However, in fatty liver disease due to insulin resistance, that is, NAFLD, with concomitant moderate alcohol consumption this may not be the case. In fact, it has been reported that alcohol-related fibrosis may progress in the absence of steatohepatitis in some cases with steatosis and perivenular fibrosis. The mechanisms of fibrogenesis in this setting are poorly characterized.^42^

The main strength of our study is that the cohort is well-characterized and with a mean follow-up time of over 17 years. We defined significant fibrosis progression as 2 or more stages of fibrosis progression to minimize sampling bias. Ratziu et al^43^ showed that when obtaining 2 samples during a liver biopsy in NAFLD patients, the probability of 1 stage or more difference upon histological evaluation was 41%. However, the probability of 2 stages or more difference was significantly lower (12%). Another strength of our study is that alcohol consumption at follow-up was assessed with 3 different methods, including PEth in blood, which only forms in the presence of alcohol.^10^ However, there are also some limitations. Our conclusions are hampered by the small number of participants in which we were not able to examine differences based on gender. We also only included patients with a follow-up visit which could create bias toward an increased possibility of liver-related outcomes in the studied patient group. There was also a small number of lifetime abstainers, which is why our comparisons were made to a group drinking no or low amounts of alcohol (0–2.99 g/week). No data was available on the type of beverage consumed (e.g. wine, beer, or spirits). Previous studies have shown that wine drinking may be preferable to beer drinking.^28^ Moreover, a thorough assessment of alcohol consumption was only performed at follow-up. At baseline, patients were only asked to describe their alcohol consumption during a typical week. In the analysis, we have assumed that the participants’ alcohol consumption has been relatively constant over the years. To support this assumption, most of the participants that were asked this question stated that their consumption pattern had been unchanged since baseline (i.e. since their first biopsy).

In conclusion, NAFLD patients consuming moderate amounts of alcohol are at increased risk for significant fibrosis progression and development of cirrhosis-related complications. PEth can be used as a potential biochemical marker to assess harmful alcohol consumption in NAFLD. Patients reporting moderate consumption or exhibiting PEth ≥48 ng/mL should be advised to reduce alcohol consumption.

## Supplementary Material
