# Supplementary material for: Moderate alcohol consumption is associated with significant fibrosis progression in NAFLD
Source: Hepatol Commun. 2023 Jan 10;7(1):e0003. doi: 10.1097/HC9.0000000000000003 (PMC9833449; doi:10.1097/HC9.0000000000000003)
Supplement: Supplementary file 1 [file hc9-7-e0003-s001.docx]

**Supplementary Table 1**. Table of baseline characteristics of participants with no or minor changes in drinking pattern. Forty-seven participants stated no or minor changes in their drinking pattern between baseline and follow-up. Thirty-five were either not asked this question (n=29) or stated significant decrease between baseline and follow-up (n=6), *i.e.*, remainder of the cohort.

|  | No/minor changes  (n=47) | Remainder of the cohort  (n=35) | *P* value |
| --- | --- | --- | --- |
| Age (years) | 46.7 (±9.5) | 48.0 (±13.2) | 0.62 |
| Sex (% male) | 78.7 (n=37) | 62.9 (n=22) | 0.14 |
| BMI (kg/m^2^) | 27.9 (±3.3) | 28.4 (±4.6) | 0.52 |
| Diagnosis of T2DM (%) | 10.6 (n=5) | 14.3 (n=5) | 0.74 |
| Hypertension (%) | 70.2 (n=33) | 54.3 (n=19) | **0.02** |
| ALT (U/L) | 82.2 (±39.0) | 85.8 (±58.2) | 0.76 |
| AST (U/L) | 42.6 (±15.0) | 49.8 (±28.2) | 0.20 |
| AST/ALT-ratio | 0.6 (±0.1) | 0.6 (±0.2) | **<0.05** |
| ALP (U/L) | 50.4 (±19.2) | 81.0 (±47.4) | **<0.001** |
| Bilirubin (mg/dL) | 0.68 (±0.40) | 0.50 (±0.20) | 0.27 |
| Albumin (g/L) | 41.6 (±3.0) | 41.5 (±3.8) | 0.93 |
| Hemoglobin (g/L) | 147.6 (±9.9) | 146.2 (±10.0) | 0.58 |
| Platelet count (10^9^/L) | 219.0 (±48.2) | 230.5 (±59.9) | 0.36 |
| Prothrombin (INR) | 1.0 (±0.1) | 1.0 (±0.1) | 0.08 |
| Creatinine (mg/dL) | 1.04 (±0.24) | 0.96 (±0.16) | 0.11 |
| Ferritin (µg/L) | 198.9 (±166.0) | 229.7 (±179.8) | 0.45 |
| Fasting glucose (mg/dL) | 104.4 (±34.2) | 115.2 (±43.2) | 0.47 |
| Fibrosis stage (IQR) | 0 (0-1) | 1 (0.25-2) | **0.01** |
| Steatosis grade (IQR) | 3 (2-3) | 2 (1-3) | 0.18 |
| Lobular inflammation (IQR) | 0 (0-0) | 0 (0-1) | **0.02** |
| Ballooning (IQR) | 0 (0-0) | 0 (0-1) | 0.06 |
| NASH (%) | 0.0 (n=0) | 20.0 (n=7) | **<0.001** |
| Significant fibrosis progression (%) | 34.0 (n=16) | 17.1 (n=6) | 0.13 |

Abbreviations: BMI, body mass index; T2DM, type 2 diabetes mellitus; ALT, alanine aminotransferase; AST, aspartate aminotransferase; AST/ALT-ratio; aspartate aminotransferase/alanine aminotransferase ratio; ALP, alkaline phosphatase; INR, international normalized ratio; NASH, non-alcoholic steatohepatitis.
